# The dramaturgy of a health emergency: Women’s lived experiences of the Zika epidemic in Brazil

**DOI:** 10.1590/1413-81232026318.00582025

**Published:** 2025-07

**Authors:** Debora Diniz, Luciana Brito, Arbel Griner, Patricia Kingori

**Keywords:** Zika epidemic, health emergencies, women, temporalities, Brazil

## Abstract

The official calendar of a health emergency has a direct impact on affected populations, particularly at the times of beginning, peak, end, and after the end. According to the World Health Organization, the Zika virus epidemic was a public health emergency between February and November 2016. The disease is now classified as an endemic arbovirus in several countries in the Global South. In this article, we analyze the chronology of the epidemic for three women affected by Zika in Alagoas, Brazil, who became pregnant at the beginning of the health emergency and whose children died between two and four years after the end of the epidemic. Our focus on the lived experience of these three women complexifies the linear dramaturgy of time in health emergencies. We aim to demonstrate how the health emergency calendar is an artifact that shapes political subjectivities, but also how it simplifies the lived experience of affected women, particularly in the times after the end.

## Introduction

In February 2016, the Zika virus was announced as a global health emergency for women of reproductive age. The emergency was declared by the World Health Organization (WHO) and attention was focused on women from a region poorly known on the international scene –the backlands of the northeastern Brazil, a vast territory where the country was formed by sugar plantations and the enslavement of people of African and Indigenous descent. It was there, where Brazil is most unequal, that the world epicenter of the Zika epidemic was made for nine months for the WHO, for eighteen months for the Brazilian Ministry of Health, and for a chronology embodied in the lived experience for the women infected by a tropical disease of everyday life, whose children were born with congenital Zika syndrome.

The Zika virus was unknown in Brazil, but it was added to the large and diverse population of local arboviruses. Dengue fever, yellow fever and chikungunya were also found in the lands of the Zika epicenter. In the second half of 2015, it seemed as if women were coming down with yet another virus or an allergy, as they had spots on their skin and a mild malaise. Some of them did not even report getting sick during pregnancy. They were experienced in tropical diseases and used to the mosquito as a common inhabitant of their daily lives.^1^ The surprise was the small-headed newborns, first described as fetal microcephaly and later as congenital Zika syndrome. A previously unknown risk to biomedicine, Zika is also transmitted vertically from the pregnant person to the fetus or through sexual relations with partners infected with the virus.^2^

A health emergency is narrated as having temporal milestones of beginning, peak and end. The chronology is imagined, as Charles Rosenberg described it, in a "dramaturgy" by those who have the authority to determine the timing of emergencies –in Brazil, the Ministry of Health, and, globally, the WHO.^3^ The time of onset is marked by the magnitude of the impact on the population, whether due to the novelty of the disease, the risk of lethality, or its contagious. The time of beginning also signals the urgency of the responses, whether to prevent the spread of the disease, to care for the sick or to seek solutions, such as research, vaccines or treatments. Authors voice some critics to the linear dramaturgy.^4^,^5^,^6^ There are health emergencies that have started late, as in the case of Covid-19, while others have had both a delayed start and a controversial end, as in the case of Ebola.^7^,^8^,^9^

With the announcement of the end of the Zika epidemic by WHO, the dramaturgy has lost the imagery attraction of graphs with numbers of infected women and photographs of children with small heads. The bulletins monitoring the epidemiological situation of Zika infection or suspected cases of congenital Zika syndrome in Brazil have become rare: the data that used to be released weekly or monthly are now compiled every six months or almost annually. In epidemiological terms, Zika has become an endemic disease in a country full of arboviruses that afflict women of reproductive age.^10^,^11^

Between 2015 and 2023 more than 22,000 children were reported with congenital Zika virus syndrome, of which 3,742 were confirmed and 2,877 are still under investigation. The remaining cases are considered inconclusive, discarded or excluded.^12^ In 2024, 539 cases were reported as suspected, of which 12 had a positive laboratory diagnosis for Zika. In 2024, more than 11,000 women of reproductive age were reported as infected with the Zika virus.^13^ This has created a time of silent continuity that biomedicine refers to as the reality of "neglected diseases".^14^ In addition, new health emergency calendars have been announced and the Covid-19 pandemic has become the world's dramaturgy. Zika has become a disease of affliction for women from a distant territory or from an older past in global health calendars.

The Zika epidemic had conflicting temporalities for women and for health policies: a woman falling ill with Zika during pregnancy was a biopolitical event if the child was affected, particularly with signs of microcephaly at birth. Otherwise, the incident of a pregnant woman falling ill passed as irrelevant to the epidemic calendar, as seem to have been the cases of women infected by the Zika virus who suffered a miscarriage (or now repeated with the oropouche virus), whose stories have disappeared from the chronology of the epidemic. Without the affected children, the women's illness was an unanswered incident in the epidemic calendar, demonstrating the complexity of telling the story of the Zika epidemic from the perspective of the time lived by the women and not just the women intertwined with their affected children.

Our research team has conducted ethnographic fieldwork with hundreds of women, from a time before the official emergency began in Alagoas to the after the end of the official calendar of the Zika epidemic in Brazil.^15^ In this article, we present the temporalities experienced by three women whose children, born at the peak of the official calendar, died between two and four years after the end of the epidemic. We seek to understand how the temporalities of motherhood and politics are intertwined with the dramaturgy of emergencies. The experience of grief was used to anchor the end of the period of care.

## Methodology and ethics

In 2016, even before the Brazilian government mapped the extent of the epidemic in Brazil, our research team traveled around the state of Alagoas in search of women and newborns who had been as affected by the Zika virus. We interviewed fifty-four women in their homes, and from this map we began national and local work to support the creation of family associations and published recommendations for public policies to protect women and children.^16^ We worked to build associations in the states of Alagoas and Rio de Janeiro, and since then we have maintained an ongoing relationship with the women and their families.

In Alagoas, the ethnographic work was combined with legal advocacy –with the Associação Família dos Anjos (AFAEAL), we work in partnership with the Judiciary for access to specialized or permanent health services, such as home care; for access to social benefits, such as income transfers and public housing. In Alagoas, thirty-two women moved to live as neighbors in housing complexes as part of the Minha Casa Minha Vida project (the result of the Association's political action), in a communal, political and affective experiment to create new zones of life during the covid-19 pandemic. Two of the three women in this article live in the same community.

AFAEAL has seventy-four registered families of children with congenital Zika syndrome. Of these, fourteen were born after the WHO declared the end of the epidemic and, of the total number of children in the Association, twelve have died. We have visited almost all of the families at their houses, participated in celebrations, public events and advocacy activities. We have built an extensive archive of interviews, documents, court cases and medical records, photographs and films. For this article, we conducted home visits and online chats via WhatsApp to explore the chronologies from the pregnancy to the deaths of the sons and daughter. The transcripts were reviewed by the women, which led to new conversations. The interviews were conducted individually, and in two cases, the partners, who were also the parents of the deceased children, participated in some of the interactions. Their narratives were complementary to those of the women but were not used as analytical material for this article.

The ethnographic accompaniment was permeated by disruptive events, such as the children's prolonged hospitalizations, the covid-19 pandemic or mourning the loss of a child. Our presence was characterized as accompaniment-witness.^17^,^18^ One of the authors of this article is a clinical psychologist, and in incidents suggesting mental distress, immediate care responses were offered, and the women were referred to long-term mental health care. We believe that the long-term bonds of trust and belonging between the research team and women allowed the accompaniment to be part of an authorized coexistence cultivated by both parties. As Ana Carla, one of the three women whose story is told in this article, said, "Your testimony is like a memory of what I experienced with Mikael and other women from the Association".

In qualitative research, anonymity of participants is a form of protection against stigma or adverse effects. We believe that care for intimacy and respect for self-image should guide ethnographic research and writing. However, as we have discussed in other contexts, not all ethnographic research assumes that anonymizing is protective; there are instances in which not naming disappears people's lived experience.^19^ In agreement with the women, this article presents them by name and image: we selected the photographs with them, and they were the first to review the text and with whom we discussed our arguments. The framing of the photographs was also agreed with each woman individually and with all of them collectively. Together we corrected facts, memories and perspectives.

The authors are fully responsible for the arguments.

The research was reviewed and approved by the Human Sciences Research Ethics Committee of the University of Brasilia under the CAAE number: 63604016.4.0000.5540. Methodologically, we used long-term ethnographic research to follow the lived experiences of the time since the woman fell ill with Zika until to the present day. We listened to the women tell their own stories, and interaction in the form of scripted questions was rare. In these years of fieldwork, there have been different forms of informed consent and image authorization. For this article, there are written informed consent for the conversations, article and photographs.

The three women are Ana Carla da Silva, Dayane Alves and Cleidiane Cavalcante. They are Northeasters, Black, young, and have interrupted their studies or precarious paid work to take full care of their children. They were beneficiaries of income transfer programs and users of the public health system. We agreed that a short chronicle would present the chronology from the time they became ill with Zika to the time after the death of their sons and daughter. The writing of the chronicle was participatory and is an experiment at feminist ethnography in which we write about and with women.^20^ In writing about each woman's experience, we used the verb temporalities in which each woman described mothering her deceased child. Some women present themselves in the present tense as "I am a mother", others as "I was a mother"; or "I have two children" or "I had a daughter and no more children". In the section "The calendars", we follow the intertwining of the dramaturgy of lived time through the categories "Incidents", "Interpretations", "Responses" and "After the end". We return to the elements of the chronicle and add other fragments of conversations about times and chronologies, indicating the voice of each woman.

### The three women and the children

Ana Carla is married to Paulo Henrique and is the mother of Miquéias Henrique, 13, and Mikael, who died at the age of 6. She was 22 when she felt ill in the third month of her pregnancy. The red spots covered her body and burned in the heat at night. "I had spots on every corner of my skin, except on my head," a topography different from that of her son Mikael's congenital Zika syndrome: in her, the disease was quick and superficial on the body; in his, it was permanent and deep, marked by white spots on the brain reduced "to a blade". Because of the spots on her skin, Ana Carla went to the doctor twice, who prescribed an ointment for allergies and to treat what was diagnosed as scabies. The spots lasted three days. As the pregnancy progressed, the television news began to show children with small heads and stories of women getting sick with spots on their bodies. Susan Sontag also explores the metaphors of the topography of illness, in this case, the topography of cancer and tuberculosis.^21^ Zika was a new disease for a woman who was born and lived surrounded by mosquitoes in a rural settlement on the Northern coast of Alagoas. In the last of three ultrasounds, she had when she was eight months pregnant, the doctor was silent as he positioned the machine on the fetus's head. Ana Carla and her husband do not know what the doctor saw. The report made no mention of the child's microcephaly. At birth, Mikael was immediately sent to the ICU. The health care team recommended that Ana Carla "not attach to her son", because his chances of survival were remote. As long as he lived, Mikael was kept in intensive care at home or in the hospital, and the family rejected anyone who "gave their son time to live". The family keeps several objects of Mikael, but the cap he used the last time has a special meaning ("it has still his smell"). Ana Carla gave birth to Mikael on February 6, 2016, and buried him on February 5, 2022.

Dayane is the mother of Maria Cecília, 4, and Emerson, who died at the age of 2. She was 18 when her husband developed Zika in the third month of her pregnancy. With little information available, Dayane was told about the importance of using insect repellent during pregnancy but was unaware of sexual transmission. She had no symptoms of Zika, and none of her prenatal ultrasounds showed any changes in fetal development. When Emerson was born, "he just had a small head". In the urban favela where she still lives, in Maceió, the capital of Alagoas, there have been no other cases of Zika or other "special children", which has generated a lot of curiosity in the community. After Emerson's death, Dayane became pregnant again during the Covid-19 pandemic, despite advice from her family and neighbors about the risk of a new child being affected by Zika or the new virus. It was as if Emerson's Zika genesis was updated with each new pregnancy or public health emergency. Doctors did not offer answers to Dayane's concerns, saying they knew little about the effects of sexual transmission of Zika and how long it remained in the body. The uncertainty of a new pregnancy made it difficult for Dayane to bond with her daughter after she gave birth and separated from her partner. Dayane is the only one of the three women who is a single mother and does not live in the housing complex, though she is still active in the WhatsApp group of other women in the Association. She keeps Emerson's maternity clothes. Dayane gave birth to Emerson on December 8, 2016, and buried him on May 28, 2019.

Cleidiane is married to Eder and was the mother of Katlyn, who died when she was six. She has no other children and is trying to get pregnant. She was 19 when she felt will in the first months of her pregnancy. She had red spots on her body when "did not even know I was pregnant", despite having a menstrual delay of more than three months. During an ultrasound, the doctor told her that "the little body didn't match the size of the brain", because the fetus' head was much smaller than expected for almost six months of pregnancy. At the same appointment where the baby was diagnosed with microcephaly, she also found out that the baby would be a girl. During her pregnancy, she was referred to a specialized rehabilitation center. The diagnosis saddened her, and she considered giving the baby up for adoption. Katlyn was born prematurely, so small that she "fit in a shoebox". Looking at her transformed the affection she felt, "she was a piece of me. I wanted to take great care of her, and I gave her a lot of love". Our first conversation with Cleidiane took place shortly after she gave birth, which left her fragile enough to participate in the research, and her partner was the main interlocutor. In subsequent conversations, she was the narrator of her and Katlyn's story. For her, the death of her daughter was the end of a cycle of love and care, which is why she insists on saying that "I would rather not dream about her"; "It ended with death, and I don't want to have any memories". Motherhood is an experience of the past, "I was a mother once, but today I don't have children". She keeps nothing from Katlyn, the pictures are with the father. Cleidiane gave birth to Katlyn on February 21, 2016, and buried her on August 15, 2022.

## The calendars

### Incidents

In Rosenberg's dramaturgy, "the peculiar texture of any epidemic reflects the continuous interaction between incident, perception, interpretation and response".3 The spots on Ana Carla and Cleidiane's skin, a disease with a superficial and ordinary topography on their bodies, were incidents considered irrelevant by the health care services and, after the symptoms disappeared, were also ignored by the women. With the birth of the children with microcephaly and the biomedical interpretation of the causality of the vertical transmission, the incident gained a new perception.^22^ But if the incident, perception, and interpretation altered the timing of the beginning of the lived experience –the birth of the child as the causal event for the risks of Zika in pregnancy –the women did not make this linear and retroactive chronology the only interpretation to situate their lived experience in time.

The timing of the epidemic coincided with the time the women's illness from a historical perspective, but not with their lived experience. The three women fell ill in the first months of the epidemic, according to the WHO, or in the prologue to the announcement of its beginning. It is only in retrospect and in the political perspective of guaranteeing rights that the women recognize themselves as "sick with Zika" at the time of the outbreak. For them, the beginning of the epidemic affliction was the ultrasound, as it was for Cleidiane, or the delivery, when they are described as mothers of a "special child". When causalities are reconstituted, calendars intertwine, and the bodies of women and children are interpreted by the science of Zika as a single subject for analysis.^23^

Even with the declaration of the global emergency that marked the time of the outbreak, there was a normalization of bodies already accustomed to being afflicted by mosquitoes or tropical diseases: they would be like ordinary incidents of life in that environmental, racial, and class geography. The three women were not diagnosed with Zika during pregnancy; Zika was a disease in the news, not imagined in their own bodies. They lived through the months of pregnancy during what has been described as the peak of the epidemic's dramaturgy in Brazil, and not even with ultrasound images suggesting fetal microcephaly, as was the case with Ana Carla and Cleidiane, was the Zika hypothesis considered by doctors.

A disquieting aesthetic tension existed between what the disease was in the women's bodies and what it manifested in the children's genesis: for the former, it was a rapid and acute topography; for the children, a permanent, chronic and deep scar. It was only with the overlapping of time for the construction of the newborn's "clinical history" that the ordinary illness of pregnancy was re-signified as Zika: the three women experienced their afflictions as nameless viruses, skin diseases, or pregnancy complications. It is the genesis of the child crossed by the "congenital Zika syndrome" that demarcates the creation of a continuous time for the three women: that of the domestic clinic of motherhood that allows the child to survive, indifferent to the uncertainties or pessimism of science.

### Interpretations

The event perceived by Ana Carla's husband, in which the doctor avoided showing Mikael's head on the ultrasound screen, suggests a discursive conflict that accompanied the peak of the health emergency. There was an excess of public circulation of images and narratives, but a discursive containment in the clinical care of women –it is as if the scientific uncertainties about the effects of the Zika virus prevented the accommodation of the common needs of women at the puerperium. Ana Carla did not meet Mikael until he was three days old and was forbidden to breastfeed him; Dayane left the maternity hospital without any information about the implications of Emerson's microcephaly; Cleidiane experienced acute psychological distress after giving birth. In contrast to the public intensity of communication, the three women faced the suspension of words and future time by the health teams. In a biographical novel about mothering a child with multiple disabilities and extreme dependency, Ada d'Adamo describes the rule of silence as "the great exit".^24^

Dayane was pregnant with her second child when the numbers of maternal deaths from Covid-19 hit Brazil: her two pregnancies followed the calendars of the country's health emergencies. Emerson's death did not mean the "end" of Zika time, as Dayane felt uncertain about the temporality of Zika through sexual transmission. In addition to the risks of Covid-19 for pregnant women, she rejected the certainties of time described by biomedicine. The doctors told her about the "six-month" window of risk after her husband became ill, but she did not believe in such temporal precision for a virus that had been unknown to science until recently.

Ana Carla offered a category to explain how silence, time and uncertainty intersected in Mikael's birth. Mikael was the newborn with the most severe congenital Zika syndrome of the Association in Alagoas (and the first child to have free public access to home care services): "People gave my son time to live". "Giving time" meant calculating survival, estimating imminent death, which would require taking Ana Carla away from her son, preventing her from breastfeeding, for example. "Give him time" came to mean Mikael's prognosis of a short life, which is why Ana Carla would hear from neighbors or family members not to "get attached" to the child. The advice was like a protective order against imminent grief.

"Creating attachment" is an expression familiar to us and to the three women and can be described as common in the Brazilian culture of affection and care. Ana Carla reminded us of how it is used to describe the relationship between humans and non-humans: the short life cycle of a domestic animal is weighed against the decision to adopt it into a family, since a shorter life span than humans would lead to a projection of suffering through death. By "giving Mikael time", the health care team kept quiet about his condition or even made it difficult for Ana Carla and her husband to get close to the child. "I had to explain to them that it was very easy for them to tell me: your son is special. I wanted them to stop giving Mikael time to live. What were they afraid of? That I would abandon him? That I would suffer?

Ana Carla refused the emptiness of time and the silence that the health care teams imposed on her son –so she took on intensive care outside the control of the hospital. Together with the AFAEAL association, Ana Carla began the dispute about who would take care of Mikael and where –at home or in a hospital intensive care unit. With other women, a domestic science for Zika was set in motion, which we call as "the clinic of the motherhood", and an intense exchange of information through online WhatsApp groups. The course of congenital Zika syndrome was unknown to science, and it was the women who were the first to identify, document and share the first convulsions, heart failures, or tube-feeding techniques. The search for the health care services took place after the domestic science had been verified, a knowledge produced by each woman in the daily practice of the clinic of the motherhood with her child.

The three women recall different situations in which their children were "disbelieved" by the medical professionals, i.e. considered to have no chance of survival. They were the ones who believed in their will to survive and gave them back the time of their lives. Ana Carla recalls three situations in which the home care staff left her son to die, and she was the one who insisted on taking care of him. The resuscitation techniques she used to save Mikael were learned from virtual classes on YouTube or from other women in WhatsApp groups. It was in the experience of the continuous time of caring that the communal living space facilitated the creation of another time and space for survival, especially with the intertwining of the Covid-19 pandemic. Regarding the importance of communal living space, one of the Association's demands was for priority access to the social housing offered by the federal government in the Minha Casa, Minha Vida project. Thirty-four women affected by Zika received free housing and are neighbors in the same urban housing community. Among the rules for receiving the housing benefit is that the beneficiary must live in the house and cannot sell it for a period of ten years.

The house was already a space of confinement before the social distancing rules of the Covid-19 pandemic. Limited accessibility for wheelchair users is a reality in peripheral urban neighborhoods in Brazil, and it is no different in the community housing buildings where the women live. Even though the houses are located on the first floor to make it easier for wheelchair users or for children to move around on their laps, a new geography of care was put into practice. The three children's so-called "pre-existing respiratory condition," which is common in children with congenital Zika syndrome, meant that the living space was reduced to the bedroom of the house to avoid the risk of Covid-19 infection.

Ana Carla, Dayane and Cleidiane described the Covid-19 months as a time of resistance to hospitalization. Every child's respiratory complication, common to being dependent on the medical devices, was assumed by the health care teams to be a potential Covid-19 infection, even though no tests were performed to confirm the disease. The children were hospitalized only in severe clinical conditions, as the women anticipated that without the domestic clinic of motherhood, the children's survival could be shortened: "It seemed that Mikael had no right to a bed during the pandemic," explained Ana Carla.

### Answers

It is in the position of political subject that the three women use the official chronology to rights advocacy: they are victims of the Zika epidemic, and their children are "special". The beginning and the peak of the health emergency coincided with the pregnancy and the birth of the children. In the political sphere, women contest the chronology of the end –the end determined by national and global health policies does not mean the end of lived experience or life needs, on the contrary. It is "after the end" of the official calendar that the lived experience of mothering a "special child" is extended.

If the language of "special child" is considered outdated for the social movements of people with disabilities in Brazil, this is not the case for the women of the Association. "Special" is not a qualifier for the child, it is part of their ontology. The compound "special because of Zika" is the inscription of the genesis and permanence of the epidemic's effects on women and children. In this way, the official calendar is strategically adopted by the women for political advocacy –the beginning of the emergency is a dramaturgy that overalps the official calendar and the lived experience, but the end is a contested temporality.

The women adopted the epidemic calendar to situate the life needs of their special children: the special condition was caused by Zika. The epidemic calendar operated as a frame of power for negotiating women's political subjectivity. "Special doesn't mean better than anyone else, much less privileged. It's just that she's different, and Zika caused it," Cleidiane explained. Being a mother of a special child positions women in the epidemic calendar but also brings them closer to other women with atypical motherhood, that is, with different times and challenges to care in a disabling social order.^25^

For all three women, it was the domestic clinic of motherhood that kept their children alive. The hospital was described as a place where "special children are rejected", where women are alienated from care. As they recounted the deaths of their children, the three women explained that a "medical error" for Dayane, a "medical negligence" for Cleidiane, and "neglect" for Ana Carla accelerated the deaths of their children. The children did not die from a natural life cycle of congenital Zika syndrome; for them, there was no natural "end" when the children were under the domestic clinic of motherhood. Mikael, Emerson and Katlyn died at an accelerated end time because they were "special children" in the hospital, according to the three women.

The end of the epidemic calendar was a time for the constitution of the political subjectivity of women – the "mothers of Zika", as they are called– and the creation of dozens of women's associations dedicated to defending their rights. The Alagoas Association is one of the most active in Brazil and was a leading voice in the process of including Zika-affected children in the main cash benefit transfer program for the extreme poor people in Brazil (Continuous Cash Benefit, BPC) and in the draft of a federal law for financial reparations for children affected by the Zika epidemic. The three children were beneficiaries of the BPC, which provides a minimum monthly wage (approximately U$300). In order to enroll the children in the BPC program, the women could not engage in paid work so that the family income threshold would not change beyond the poverty line. In addition, the children required intensive and continuous care. When the children died, the benefit was suspended. The financial impact on the families was felt directly by the women, whose paid work plans were interrupted by years of caregiving. At the beginning and peak of the epidemic in 2016, conversations about financial reparations for the impact of Zika on women's life plans and children's health were ignored: Ana Carla felt that "it seems like I'm rejecting my child". It was only in the "after the end", with the actions of different associations in the country and the political projection of women, that the law on reparations for Zika children started to be discussed. In addition, the restrictive interpretation that the cash transfer is for the disabled child and not also for the caregiver has placed women in an even more precarious situation of poverty. The discussion about the lack of protection experienced by caregivers of children with disabilities or people affected by health emergencies is on the agenda of political negotiations in Brazil, with the Association playing a strong role.

Bill 6064/23 was vetoed by the federal government and sent back to the Senate. The bill reconsiders children as subjects of the right to reparations, excluding women. The bill creates a new epidemic calendar for the purposes of reparations: children born between 2015 and 2019. For Ana Carla, Cleidiane and Dayane, reparations for children are considered fair, not only because there is no separation between what happened to the children and what happened to them. However, the assumption that the child must be alive in order to be entitled to reparations is contested. The value of the reparation is estimated in U$10,000 for each child, which has a significant impact for the families. Recognizing the right to reparations for Mikael, Emerson and Katlyn is more than a financial claim about the preventable epidemic impact on their children; it is above all a political identification of the three women in the historical moment of the Zika epidemic in Brazil. It is also about Zika affected women and their lived experiences: "reparations", they say, "are about what happened to us and our children, and it doesn't matter when they were born or died". In other words, there is a demand that the effects of the epidemic calendar remain in place, even for women whose children have already died, to whom an undeniable "end" could be projected, or for children born after the end of the reparation calendar of the bill.

### After the end

Ethnographic research has allowed us to problematize how the epidemic calendar shapes political subjectivities for political advocacy or financial reparations, but also how lived experience of motherhood and mourning extrapolates the linearity of the dramaturgy of emergencies. The times of the dramaturgy are political events and go beyond the epidemiological analysis of risks and emergencies based on the biology of diseases or the population's need for care. In the case of the Zika epidemic, the emergency official calendar did not begin when the first pregnant woman fell ill with an arbovirus unknown in Brazil; when the first newborn with microcephaly was born; or when science published a paper demonstrating vertical transmission of Zika. It was only after the emergency calendar was put in place that biopolitical time could coincide with the time experienced by the first communities and people affected. In other words, it is with the official calendar that the people of a population come to exist politically as subjects with needs that is legitimized and recognized by the political power.

Dramaturgy is a narrative framework in which different elements are combined to delineate the short, long, interrupted, or cyclical periods of a health emergency. The dramaturgy signals that a disease has reached the biopolitical scale of a pandemic or epidemic, that it has become endemic, or that it has been eradicated. A disease's place in the dramatic narrative moves financial resources, academic research and news; national and global public health policies; affections and behaviors such as mourning, fear, xenophobia, or solidarity. However, there is a growing body of analysis that suggests that the dramaturgy framework is inadequate for understanding the impact of health emergencies on different countries, communities, or affected people.

If the times of a health emergency call on people exposed to the risk of disease to locate themselves in the chronology of the beginning, the peak, or the end, the ways of creating and living subjectivities about the time lived are not linear as the framework of dramaturgy imagines. In the case of the Zika epidemic in Brazil, the women's times sometimes dialogue with the official chronology, sometimes ignore it, or even reject it. With this study, we hope to have contributed to a growing research agenda on temporalities and health emergencies, particularly on the meanings experienced "after the end" for the people and communities affected.
